# TIG3 Tumor Suppressor-Dependent Organelle Redistribution and Apoptosis in Skin Cancer Cells

**DOI:** 10.1371/journal.pone.0023230

**Published:** 2011-08-17

**Authors:** Tiffany M. Scharadin, Haibing Jiang, Ralph Jans, Ellen A. Rorke, Richard L. Eckert

**Affiliations:** 1 Department of Biochemistry and Molecular Biology, School of Medicine, University of Maryland, Baltimore, Maryland, United States of America; 2 Department of Dermatology, School of Medicine, University of Maryland, Baltimore, Maryland, United States of America; 3 Department of Obstetrics and Gynecology and Reproductive Sciences, School of Medicine, University of Maryland, Baltimore, Maryland, United States of America; 4 Department of Microbiology and Immunology, School of Medicine, University of Maryland, Baltimore, Maryland, United States of America; University of Medicine and Dentistry of New Jersey, United States of America

## Abstract

TIG3 is a tumor suppressor protein that limits keratinocyte survival during normal differentiation. It is also important in cancer, as TIG3 level is reduced in tumors and in skin cancer cell lines, suggesting that loss of expression may be required for cancer cell survival. An important goal is identifying how TIG3 limits cell survival. In the present study we show that TIG3 expression in epidermal squamous cell carcinoma SCC-13 cells reduces cell proliferation and promotes morphological and biochemical apoptosis. To identify the mechanism that drives these changes, we demonstrate that TIG3 localizes near the centrosome and that pericentrosomal accumulation of TIG3 alters microtubule and microfilament organization and organelle distribution. Organelle accumulation at the centrosome is a hallmark of apoptosis and we demonstrate that TIG3 promotes pericentrosomal organelle accumulation. These changes are associated with reduced cyclin D1, cyclin E and cyclin A, and increased p21 level. In addition, Bax level is increased and Bcl-XL level is reduced, and cleavage of procaspase 3, procaspase 9 and PARP is enhanced. We propose that pericentrosomal localization of TIG3 is a key event that results in microtubule and microfilament redistribution and pericentrosomal organelle clustering and that leads to cancer cell apoptosis.

## Introduction

TIG3 (Tazarotene-induced gene 3), which is also called retinoic acid receptor responder 3 (RARRES3) and retinoid-inducible gene 1 (RIG1) [1]–[5], is a one hundred sixty-four amino acid protein [6]. TIG3 was originally identified as increased following treatment of cultured epidermal keratinocytes or psoriatic epidermis with the synthetic retinoid, Tazarotene [6]. It is expressed at low levels in hyperproliferative epidermis (e.g., squamous cell carcinoma and psoriasis) and expression is restored by retinoid treatment [7]–[9]. In retinoid-treated psoriatic epidermis, increased TIG3 expression is associated with restoration of normal differentiation [6], [10]. The association of increased TIG3 expression with normal epidermal phenotype suggests that TIG3 may act as a pro-differentiation regulator. To examine the mechanism of action, we studied TIG3 function in normal human keratinocytes [10]–[12]. These studies show that TIG3 is present at vanishingly low levels in keratinocytes in monolayer culture, but is increased in differentiated raft cultures [12]. Vector-mediated expression of TIG3 in keratinocytes results in reduced proliferation and increased cornified envelope formation, suggesting that TIG3 regulates keratinocyte differentiation [10]–[12]. Ongoing studies show that TIG3 operates via several mechanisms, but a prominent mechanism of action is regulation of transglutaminase activity [10], [11]. Type I transglutaminase (TG1) is a key enzyme in keratinocytes and other surface epithelia that is expressed in suprabasal differentiated cells [13]–[20]. Transglutaminase catalyzes formation of ∈-(γ-glutamyl)lysine protein-protein crosslinks to assemble the cornified envelope, an essential component of the epidermal barrier [21], [22]. Our studies suggest that TIG3 co-localizes with TG1 leading to increased transglutaminase activity [10], [11]. Additional studies show that TIG3 reduces keratinocyte proliferation, but does not cause apoptosis [10], [11]. TIG3 consists of an amino terminal hydrophilic segment and a c-terminal membrane anchoring domain [6], [23]. Mutagenesis studies indicate that mutants lacking the c-terminal membrane-anchoring domain are not active [10], [11], [23]. In contrast, N-terminal truncation converts TIG3 into a protein that causes apoptosis in keratinocytes [12].

TIG3 is expressed at reduced levels in skin tumors [7]. Thus, a major goal of the present study is to characterize the impact of TIG3 expression in skin cancer cells. We show that restoring TIG3 expression reduces survival of epidermal squamous cell carcinoma cells via a mechanism that involves pericentrosomal TIG3 localization leading to altered microtubule organization and organelle distribution. This is associated with changes in the level of cell cycle and apoptosis regulators.

## Results

### TIG3 expression decreases cell number

**Figure 1:**
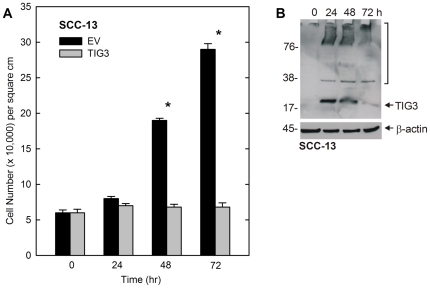
TIG3 decreases cell survival. Subconfluent cultures of SCC-13 cells, growing in 3.8 cm^2^ wells, were infected with 10 MOI tAd5-EV or tAd5-TIG3. At 0, 24, 48, and 72 h post-infection, cells were counted and lysates prepared. **A** TIG3 expression decreases cell number. Values are mean ± SEM, n = 3. Those comparisons marked by an asterisk are significantly different as determined by Student's t-test (p<0.001). **B** TIG3 is detected by immunoblot in tAd5-TIG3 infected SCC-13 cells. The monomer is visible at 18 kDa and the bracket indicates higher molecular weight crosslinked TIG3 [10], [11]. Molecular weights are indicated to the left of the blot in kDa.

We began by examining the impact of TIG3 on SCC-13 cell survival. TIG3 was delivered by adenovirus infection. **Fig. 1A** shows that empty vector-infected cells increase in number over 72 h, but that cell number is significantly reduced at 48 and 72 h in TIG3-expressing cells. **Fig. 1B** shows that TIG3 level is maximal in the infected cells by 24 and 48 h post-infection and is reduced by 72 h. In addition to the TIG3 monomer, we observe accumulation of high molecular weight forms which are thought to be covalently-crosslinked TIG3 [10]–[12]. As previously reported, TIG3 is expressed at low levels in most transformed cells [10], [11] and therefore is not detected at time zero.

### TIG3 decreases cell proliferation by inhibiting cell cycle progression

**Figure 2:**
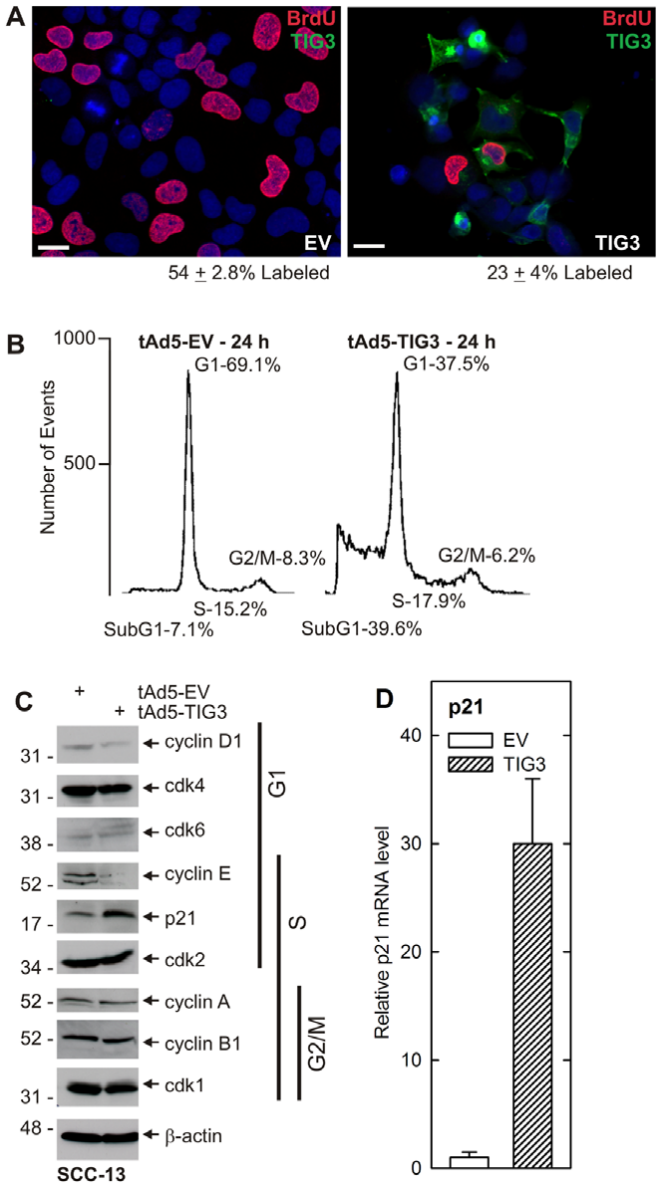
TIG3 reduces cell cycle progression. **A** SCC-13 cells grown on coverslips were infected with 10 MOI of EV or TIG3-encoding virus and after 24 h treated with 10 µM BrdU for 2 h and then fixed and stained with anti-BrdU (red) and anti-TIG3 (green). BrdU incorporation is a marker of the synthesis phase of the cell cycle. The number of BrdU positive cells as a percentage of total cell number is presented beneath each panel. The values are mean ± SEM (n = 3) and the values are significantly different as determined by Student's t-test (p<0.001). Bars = 10 µm. **B** SCC-13 cells were collected for flow cytometry at 24 h post-infection with EV or TIG3-encoding virus. Cells were stained with 50 µg/ml propidium iodide prior to analysis. TIG3 reduces events in G1 and increases subG1 events. **C** At 24 h post-infection, cells were harvested and extracts prepared for detection of cell cycle regulatory proteins. Molecular weights are indicated to the left of the blot in kDa. **D** Cells were treated as above and then harvested for detection of p21 encoding mRNA by real time-PCR. A similar result was observed in each of three experiments.

We next monitored cell cycle progression. We began by assessing the percentage of cells in S-phase using BrdU labeling. SCC-13 cells were infected with TIG3-expressing virus and after 24 h labeled with BrdU for 2 h before detection of BrdU and TIG3. As shown in **Fig. 2A**, 54±2.8% (mean ± SEM) of EV-infected cells were positive for BrdU as compared to 23%±4% of TIG3-expressing cells. As shown in **Fig. 2B**, the most prominent cell cycle changes are a reduction in G1 and increase in subG1 events. To investigate the mechanism responsible for these changes, we measured the level of key cell cycle regulatory proteins. **Fig. 2C** shows that cyclin-dependent kinase (cdk1, cdk2, cdk6, cdk4) levels are not altered by TIG3, but cyclin D1 and cyclin E levels are decreased and p21 level is increased. These changes are consistent with reduced progression through the G1 phase and the G1/S transition. **Fig. 2D** shows that p21 mRNA level increases in parallel with the increase in p21 protein.

### TIG3 induces apoptosis

**Figure 3:**
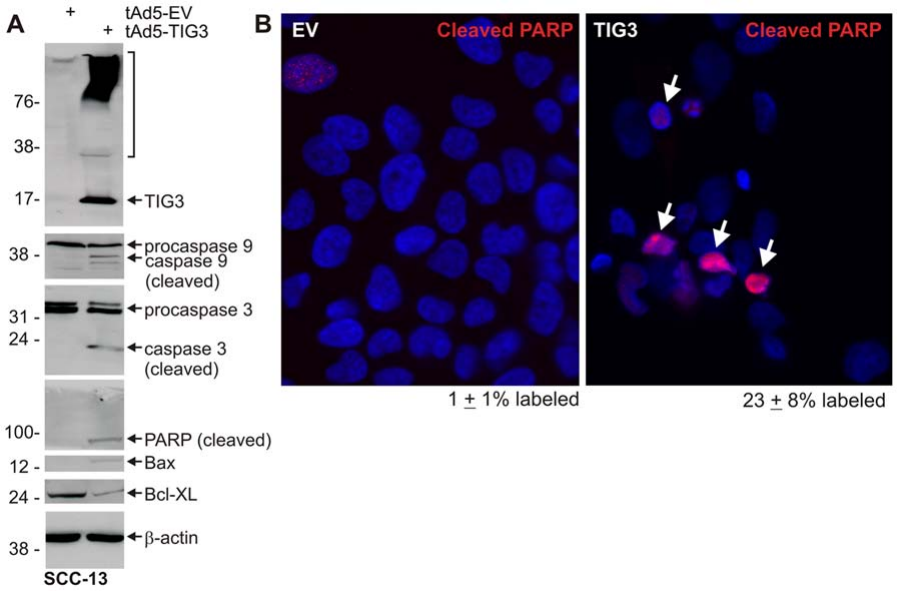
TIG3 induces apoptosis. **A** SCC-13 cells were infected with 10 MOI of EV or TIG3-encoding virus and after 24 h lysates were prepared for detection of apoptosis markers. The bracket indicates high molecular weight crosslinked TIG3 [10], [11]. Molecular weights are indicated to the left of the blot in kDa. **B** At 24 h post-infection SCC-13 cells were fixed and stained with anti-cleaved PARP (red). TIG3 increases cleaved PARP staining. The percentage of cleaved PARP positive cells is presented in each panel (mean ± SD). The arrows indicate cleaved PARP-positive cells. Similar results were observed in each of three experiments.

The presence of subG1 DNA content can be associated with cell apoptosis. We therefore assessed the impact of TIG3 on apoptosis. As shown in **Fig. 3A**, TIG3 expression activates cleavage of procaspase 9 and procaspase 3 and generates cleaved PARP. In addition, the pro-apoptotic regulator, Bax, is increased and the prosurvival regulator, Bcl-XL, is reduced. Increased apoptosis can also be observed *in situ*. **Fig. 3B** shows that very few cleaved PARP-positive cells are observed in control cultures (1±1%, mean ± SD), but that the number is markedly increased in TIG3-positive (23±8%) cultures. These findings, together with the increase in subG1 DNA content (**Fig. 2B**), suggest that TIG3 induces apoptotic cell death.

### TIG3 localizes at a pericentrosomal location and causes organelle redistribution

**Figure 4:**
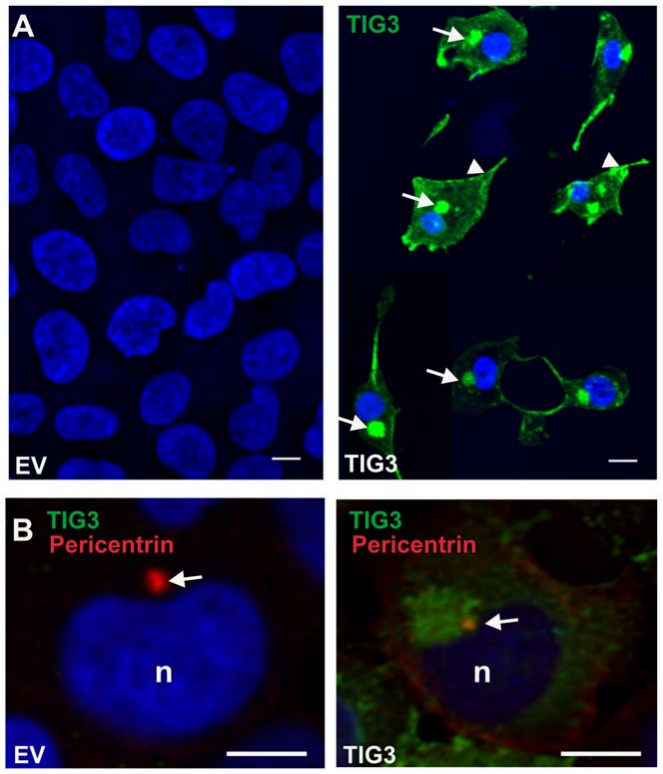
TIG3 localizes in the vicinity of pericentrin. **A** SCC-13 cells were infected with 10 MOI tAd5-EV or tAd5-TIG3 and at 24 h post-infection were fixed and stained with anti-TIG3 (green). Arrows indicate pericentrosomal and arrowheads indicate membrane localization. No TIG3 is detected in empty vector-infected cells. **B** Cells, infected as above, were fixed and stained with TIG3 (green) and pericentrin (red). The arrows indicate pericentrin staining of the centrosome and n indicates the nuclei. Bars = 10 µm.

To gain insight into the TIG3 mechanism of action we monitored TIG3 subcellular location in tAd5-TIG3 virus-infected cells. As shown in **Fig. 4A**, TIG3 (green) accumulates along the cell periphery near the plasma membrane (arrowheads) and in a perinuclear location (arrows), and the nuclei in TIG3-expressing cells are reduced in size. To further assess the TIG3 location, cells were stained to detect pericentrin, a centrosome marker. The images in **Fig. 4B** reveal TIG3 staining juxtaposed with pericentrin staining (white arrow) adjacent to the nucleus (n). TIG3 (green) localizes in the general vicinity of pericentrin (red) suggesting accumulation in the vicinity of the centrosome.

**Figure 5:**
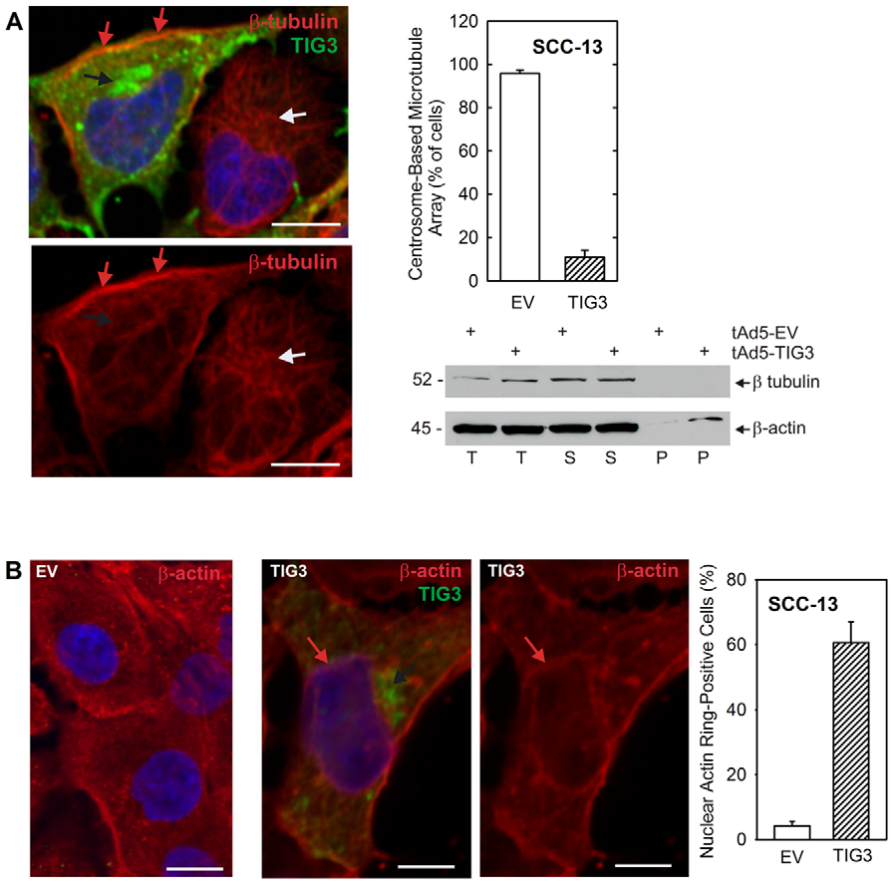
TIG3 alters microtubule distribution. **A** SCC-13 cells were infected with 10 MOI tAd5-EV or tAd5-TIG3 and at 24 h post-infection cells were fixed and stained with anti-TIG3 (green stain) and anti-β-tubulin (red stain). TIG3 accumulates at the expected perinuclear location (black arrow). β-tubulin accumulates in an atypical ring at the cell periphery (red arrows). The normal β-tubulin network in the TIG3-negative cell is indicated by a white arrow pointing to the centrosome. Nuclei were Hoechst stained (blue). The bottom panel is identical to the top, except that only the β-tubulin (red) signal is indicated. Bars = 10 µM. The graph shows the number of tAd-EV and tAd5-TIG3 infected cells with centrosome-originated microtubule arrays. Cells were counted in twenty independent microscope fields and a minimum of one-hundred cells were counted per treatment group. The values are mean ± SEM. Paired Student's t-test analysis reveals that the means are significantly different (p<0.001). To assess the impact of TIG3 on tubulin solubility, cells were infected with tAd5-EV or tAd5-TIG3 and after 24 h total extract, soluble and pellet fractions were prepared and electrophoresed followed by immunostaining to detect β-tubulin and β-actin. The presence of the majority of β-actin in the soluble fraction indicates that the fractionation was successful. **B** TIG3 expression causes actin filament collapse around the nucleus. SCC-13 cells were infected with adenovirus as above and after 24 h stained with anti-β-actin (red stain) and anti-TIG3 (green stain). Nuclei were Hoechst stained (blue). For the TIG3-positive cells, the left panel shows the TIG3 and β-actin signals (red and green), while the right panel shows only the β-actin (red) channel. The black arrow indicates TIG3 accumulation at the centrosome and the red arrow indicates the β-actin microfilament nuclear ring. Bars = 10 µm. The plot shows the number of tAd-EV and tAd5-TIG3 infected cells displaying actin microfilament rings surrounding the nucleus. Cells were counted in eighteen independent microscope fields and a minimum of one-hundred cells were counted per treatment group. The values are mean ± SEM. Paired Student's t-test analysis reveals that the means are significantly different (p<0.001).

The centrosome is a control point for a wide range of cell functions. It functions as the microtubule organizing center (MTOC) which is the site of microtubule assembly [24], [25]. In addition, it replicates during cell division and the daughter centrosomes serve to organize the mitotic spindles that guide chromosome separation [24], [26]. Importantly, the MTOC serves as a control point for movement of molecular motor-carried cargo, including organelles, along the microtubules [27]. Moreover, centrosome dysfunction is associated with cell apoptosis [28]. We therefore examined whether pericentrosomal TIG3 accumulation alters microtubule distribution. As shown in **Fig. 5A**, the microtubules in TIG3-negative cells are spread throughout the cell in a typical lattice-type network that radiates out from the centrosome (white arrow). In contrast, in TIG3-expressing cells (black arrow indicates pericentrosomal TIG3), the microtubules localize as a band (red arrows) at the cell periphery and do not radiate out from the centrosome, suggesting that TIG3 impacts microtubule location and anchorage. This is best observed in **Fig. 5A** (lower panel) which shows only the β-tubulin staining. Microtubules in the TIG3-positive cell (left) lack centrosome-based clustering, while the TIG3-negative cell (right) has centrosome-anchored microtubules (white arrow). To assess the impact of TIG3 on the microtubule network, the number of cells displaying microtubules radiating from the centrosome was counted. As shown in the plot, the percentage of cells displaying centrosome-directed networks is markedly reduced in TIG3-expressing cells.

The redistribution of tubulin to the cell membrane in TIG3-positive cells suggests that it may be insoluble. To assess this, we prepared total cell extract from TIG3-negative and positive cells and prepared soluble and particular (pellet) fractions. We then assayed for β-tubulin in each fraction by immunoblot. As shown in **Fig. 5A**, the membrane-localized β-tubulin does not appear in the pellet fraction, suggesting that although it is localized at the cell periphery at or near the membrane, it remains soluble.

The tubulin and actin networks interact extensively and so we monitored the impact of TIG3 on actin filament distribution (**Fig. 5B**). In TIG3-negative cells (EV), β-actin (red) distributes throughout the cytoplasm. In contrast, in TIG3-positive cells (green stain) a distinct actin filament ring forms at the nuclear periphery (red arrow). This is shown in both a merged image and β-actin staining alone (**Fig. 5B**). The number of cells displaying a nuclear actin ring was counted. The plot shows that the percentage of cells displaying a nuclear actin ring is markedly increased in TIG3-expressing cells.

**Figure 6:**
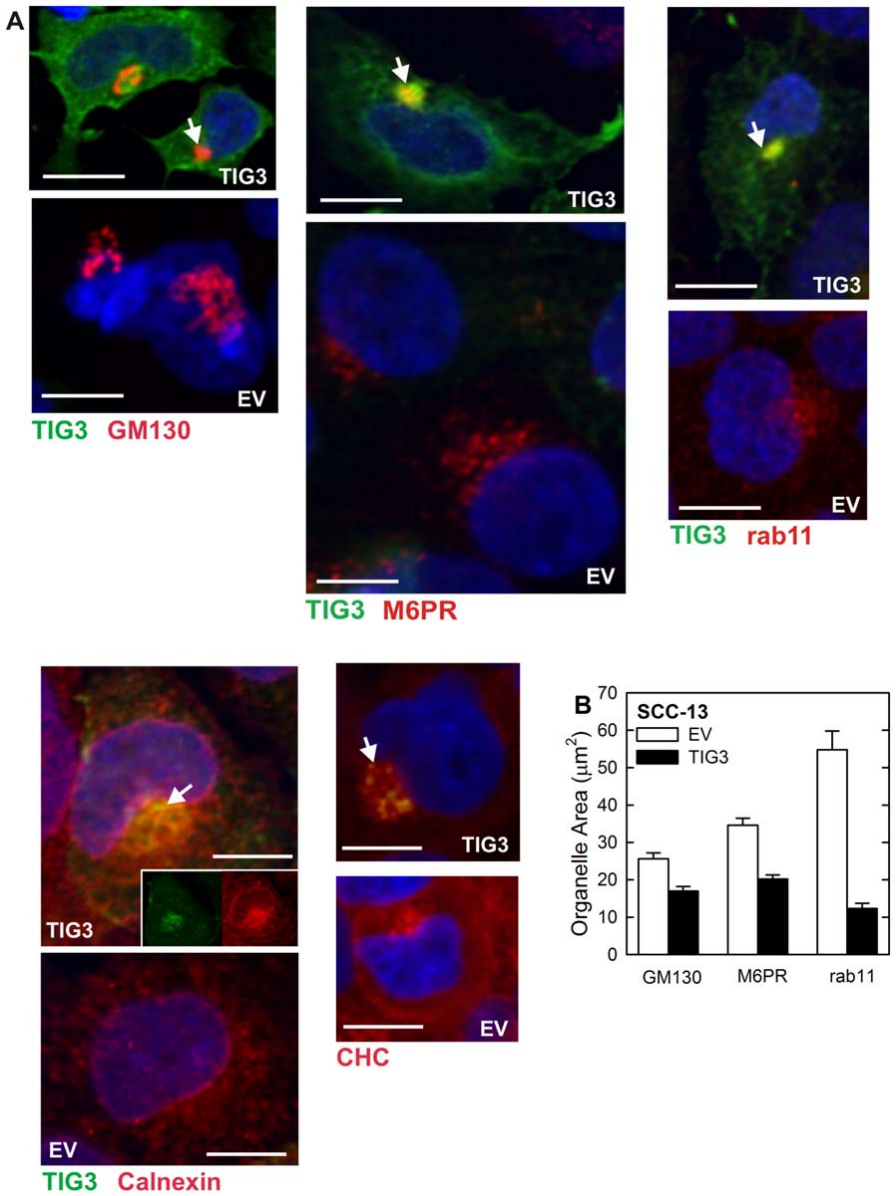
Pericentrosomal organelle accumulation in TIG3-positive cells. **A** SCC-13 cells were infected by 10 MOI tAd5-EV or tAd5-TIG3 and after 24 h fixed and stained to detect TIG3 (green) and various organelle markers (red). The markers include GM130 (cis-Golgi), M6PR (mannose-6-phosphate receptor, trans-Golgi and late endosome), rab11 (recycling endosome), calnexin (ER), and CHC (clathrin heavy chain, intracellular transport vesicle). White arrows indicate pericentrosomal localization of TIG3. Nuclei are stained blue. For GM130, M6PR, and rab11, all panels are red/green/blue merged images. The EV and TIG3 pictures of calnexin staining are red/green/blue merged images. The inserts in the TIG3/calnexin picture are single-color images. For the CHC stain, only the red (CHC) image is shown. Bars = 10 µm. **B** TIG3 impact on subcellular organelle distribution. To measure organelle spread, the microscope was focused on the z-plane displaying the maximal organelle spread and the area covered by the organelle was monitored using ImageJ Software and presented as organelle area in µm^2^. The values are mean ± SEM (n = 30 fields) including measurement of a minimum of thirty cells per treatment group. Paired Student's t-test analysis identifies the means as significantly different (p<0.001).

**Figure 7:**
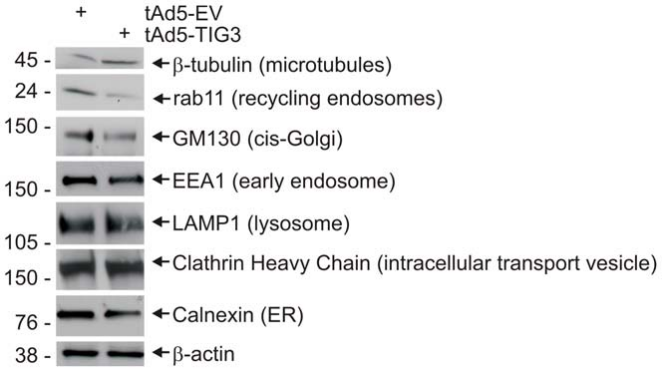
TIG3 impact on organelle marker protein level. SCC-13 cells were infected by 10 MOI tAd5-EV or tAd5-TIG3 and after 24 h cell lysates were prepared for immunoblot detection of the indicated proteins. TIG3 expression reduces the level of rab11, GM130 and EEA1, but does not alter LAMP1 level. Molecular weights are indicated to the left of the blot in kDa.

Because organelle movement and anchoring depend upon the tubulin and actin networks [29], we anticipated that TIG3 may influence organelle distribution. To assess the impact of TIG3 on organelle location, cells were stained to detect GM130 (cis-Golgi), mannose-6-phosphate receptor (M6PR, trans-Golgi and late endosome) [30], rab11 (recycling endosomes), calnexin (endoplasmic reticulum), and clathrin heavy chain (CHC, intracellular transport vesicles). **Fig. 6A** shows that GM130 (red) localizes at a perinuclear location in TIG3-negative and positive cells; however, it appears more compacted in TIG3-positive (green stain) cells. M6PR, rab11 and calnexin also appear compacted at the pericentrosomal location in TIG3-positive cells (white arrows). This is readily visualized for calnexin, by comparing the distribution shown in the red single-color image (insert) with that observed in EV-infected cells (**Fig. 6A**), as an intense pericentrosomal concentration of calnexin is observed in TIG3-positive cells. In addition, clathrin heavy chain is a particularly dramatic example, in that staining appears distributed throughout the cytoplasm in EV-infected cells, but essentially all of the CHC accumulates in the vicinity of the centrosome in TIG3-positive cells. Thus it appears that TIG3 alters intracellular organelle distribution in a manner that concentrates and compresses many organelles in the vicinity of the centrosome. This compaction was quantified by measuring the two-dimensional area covered by individual organelles in square microns using ImageJ Software (**Fig. 6B**). This analysis reveals a substantial and statistically significant reduction in area for GM130, M6PR and rab11. Calnexin and CHC distribution was not analyzed in this manner, since the condensation was obvious. We also examined the impact of TIG3 on organelle protein level. **Fig. 7** shows that TIG3 expression causes a modest reduction in the level of some organelle proteins, including calnexin, rab11, GM130 and EEA1; however, the level of other marker proteins is not altered.

## Discussion

TIG3 (Tazarotene-induced gene 3) was originally identified as increased following treatment of cultured epidermal keratinocytes or psoriatic epidermis with the synthetic retinoid, Tazarotene [6], and was later identified in gastric cancer cells and called RIG1 [5]. Subsequent studies reveal TIG3 mRNA in a range of tissues and cells in culture [1], [5], [31]–[33]. TIG3 level is increased by retinoid treatment [4], [34] and in some cell types TIG3 gene expression is suppressed by MAPK signaling [35].

TIG3 is a member of the NIpC/P60 superfamily of proteins [36]. The N-terminal domain (aa1–134) encodes regions that are conserved among members of the lecithin∶retinol acyltransferase (LRAT) and H-rev tumor suppressor families [37]–[39]. Functional analysis of the TIG3 structure indicates that the c-terminal hydrophobic domain (aa135–164) functions as a membrane anchor [6], [23] and that the N-terminal region encodes calcium-independent phospholipase A1/2 activity [31], [32], [40]. Moreover, phospholipase A1/2 activity does not require the c-terminal hydrophobic domain [40]. TIG3 has been shown to reduce cell survival in a number of cell types [6], [10], [11], [34], [41], [42], but the mechanism responsible for the suppression is not well understood. In some cell types, TIG3 may act via regulation of MAPK and PI3K/Akt signaling [1], [3], [41].

### TIG3 in epidermis

TIG3 is expressed in the suprabasal differentiated layers of keratinocyte raft cultures [12] and in suprabasal epidermis [7], [8], and TIG3 expression is reduced in hyperproliferative skin disease and in skin cancer cells [6]–[9]. Retinoid treatment increases TIG3 level and this is associated with reduced cell proliferation [6]–[9]. TIG3 is not expressed in normal keratinocytes maintained in monolayer culture and vector-mediated TIG3 expression is associated with reduced cell number [8], [10]–[12]. Mechanistic studies in keratinocytes show that the c-terminal membrane anchoring domain is required for activity [10], [11], [23], and that expression of TIG3 in normal human cultured keratinocytes activates selected differentiation-related events [10]–[12]. In particular, TIG3 interacts with type I transglutaminase (TG1) to increase TG1 catalytic activity [10], [11]. TG1 is a key enzyme in differentiating keratinocytes that catalyzes formation of protein-protein crosslinks leading to assembly of the cornified envelope, an important component of the skin permeability barrier [43]. Full-length TIG3 protein stimulates differentiation-associated transglutaminase activity in keratinocytes. In contrast, amino-terminal truncated forms cause apoptosis and the level of apoptosis is more pronounced as the N-terminus is shortened [12]. Other studies point to unique effects of various TIG3 subdomains [40], [42], [44]. The suprabasal pattern of TIG3 expression in epidermis is consistent with a role of TIG3 as a controller of keratinocyte differentiation-related events.

### TIG3 expression in skin cancer cells reduces proliferation and increases apoptosis

Previous studies indicate that TIG3 mRNA is present at reduced levels in skin cancer and in skin cancer cell lines [8]. However, little is known regarding whether TIG3 regulates skin cancer cell survival and tumor progression. We show that expression of TIG3 causes a marked reduction in SCC-13 cell number that is associated with reduced G1 and S phase events and increased sub-G1 DNA content. These cell cycle changes are associated with TIG3-dependent changes in cell cycle regulatory protein level. TIG3 expression reduces cyclin D1 and cyclin E levels and increases the level of the p21 cyclin-dependent kinase inhibitor. These findings are consistent with a reduction in cell progression through the G1/S transition. In addition, we demonstrate that TIG3 increases SCC-13 cell apoptosis as evidenced by increased production of activated caspase 9 and 3 and increased cleaved PARP. Moreover, immunostaining studies reveal cleaved PARP accumulates in TIG3-positive cells. These results are particularly interesting as TIG3 does not cause apoptosis in normal human keratinocytes. Instead, TIG3 causes the cells to undergo differentiation [10]–[12]. In contrast, mutant forms of TIG3 cause apoptosis in normal human keratinocytes [12]. The fact that TIG3 causes apoptosis in cancer cells suggests a different mechanism of action in normal versus transformed cells. In addition, some of these changes are associated with changes in target gene mRNA level. For example, the TIG3-dependent increase in p21 protein is associated with a parallel increase in p21 encoding mRNA, indicating that TIG3 regulates p21 gene transcription or RNA stability. We do not presently know whether this action is direct or indirect.

### TIG3 expression is associated with pericentrosomal organelle clustering

An important question is where TIG3 is localized in the cell. An interesting previous study in HeLa cervical cancer cells suggests that TIG3 localizes in the cis- and trans-Golgi and that this localization is required to stimulate apoptosis [2]. As shown in **Fig. 6A**, antibody co-staining of SCC-13 cells suggests that TIG3 localizes in the cis- and trans-Golgi (GM130, M6RP), the late endosome (M6PR), the recycling endosome (rab11), the endoplasmic reticulum (calnexin) and the intracellular transport vesicles (CHC). However, we believe that is it unlikely that TIG3 is principally localized in these structures for several reasons. First, TIG3 only appears to localize with the organelle only in the immediate vicinity of the centrosome and not more peripherally. Second, we show that TIG3 alters microtubule and microfilament distribution and this is associated with pericentrosomal organelle accumulation. Third, in another report we are studying the distribution of TIG3 in normal human keratinocytes and these studies strongly suggest a pericentrosomal TIG3 location (not shown). Based on these findings, we argue that the major effect of TIG3 is at the centrosome and that the appearance of colocalization of TIG3 with organelle markers is an artifact due to pericentrosomal organelle clustering.

Organelle relocation is a well-known phenomenon that occurs during apoptosis [45]. It is thought to enhance organelle membrane mixing to facilitate spread of pro-apoptotic effectors [45]–[50]. However, how these organelles and organelle fragments come together during apoptosis it is not well understood, but is thought to involve the centrosome and microtubules. The centrosome serves as a microtubule organizing center (MTOC) in interphase cells and as an organizer of the mitotic apparatus in mitotic cells. As a microtubule nucleation center, centrosome function is required for intracellular organelle trafficking [24], [51]. Organelles move along microtubules associated with specific motor proteins [27], [51]. Previous reports implicate microtubule motors in bringing organelle and organelle fragments to the microtubule organizing center (centrosome) during apoptosis [45], [52]. These include the Golgi apparatus, endosomes, endoplasmic reticulum, mitochondria and other organelles [45], [47], [53], [54]. The best characterized example is redistribution of mitochondria to the Golgi-proximal microtubule organizing center in cells exposed to TNFα, oxidative stress or viral infection [45]. Several reports suggest that this process activates MAPK signaling to phosphorylate kinesin light chain to halt mitochondria anterograde dispersal leading to accumulation of these organelles near the centrosome [45].

These previous reports describe pericentrosomal organelle clustering in response to treatment with growth factors, oxidative stress or other stimuli [45]. Our present studies are unique in that the organelle clustering is triggered by expression of a tumor suppressor protein. Moreover, the fact that TIG3 accumulates near the centrosome suggests that it may have a direct role in regulation of organelle movement during apoptosis. Our studies reveal that TIG3 presence alters the normal subcellular location of microtubules and microfilaments, which may be one mechanism whereby it alters organelle location. Future studies will need to address whether TIG3 also alters microtubule motor-dependent transport of organelles. Our working hypothesis is that TIG3 may alter both microtubule distribution and microtubule-based motor function to cause pericentrosomal organelle clustering. Based on our analysis, TIG3 promotes accumulation of organelles at the centrosome including endoplasmic reticulum, Golgi apparatus, recycling endosomes, late endosome, and transport vesicles. However, not all organelles are transported to the centrosome. For example, lysosomes, as measured by detection of LAMP1, distribute in arrays along microtubules and, in TIG3-positive cells, this pattern is intensified (not shown). Thus, additional studies will be necessary to understand the role of TIG3 in regulating microtubule function and organelle transport.

## Materials and Methods

### Cell Culture and adenovirus infection

SCC-13 cells were obtained from American Type Culture Collection (Rockville, MD) and were maintained in high glucose DMEM (Gibco, 11960-044) supplemented with 2 mM L-glutamine, 1 mM sodium pyruvate, 100 U/ml penicillin, 100 µg/ml streptomycin and 5% fetal bovine serum (Sigma, St. Louis, MO). Adenoviruses were produced as previously described [10]. tAd5-TIG3_1–164_, is a tetracycline-inducible virus that encodes the full-length TIG3 protein and a tetracycline-responsive enhancer element [10], [11]. The Ad5-TA virus encodes the tetracycline transactivator (TA). tAd5-EV is an empty virus used as a control. For infection, cells were washed with PBS, incubated with 10 MOI of TIG3-encoding or empty virus in the presence of 5 MOI of Ad5-TA in DMEM containing 6 µg polybrene/ml (Sigma, H9268). After 5 h, the medium was replaced with virus-free medium and incubation was continued for an additional 24–72 h prior to preparation of cells and extracts for immunohistology and immunoblot.

### Immunological methods

For immunoblot, extracts were prepared in cell lysis buffer (20 mM Tris-HCl, pH 7.5, containing 150 mM NaCl, 1 mM ethyleneglycol-bis(aminoethylether)-tetraacetic acid, 1 mM EDTA, 1% Triton X-100, 2.5 mM sodium pyrophosphate, 1 mM glycerophosphate, 1 mM sodium vanadate, 1 µg/ml leupeptin (Cell Signaling, 9803, Danvers, MA) and 1 mM phenylmethylsulfonyl fluoride. Protein concentration was determined using the Bradford Bio-Rad protein assay (Bio-Rad, 500-0006). Equal amounts of protein were electrophoresed on Ready Gels (Bio-Rad, Hercules, CA) and transferred to nitrocellulose membranes for detection of proteins using the appropriate antibodies. For immunofluorescence, cells maintained on coverslips, were infected with adenoviruses and after 24–72 h fixed in 4% paraformaldehyde, permeabilized with methanol, and incubated with rabbit anti-TIG3 (1∶100) and selected organelle marker antibodies followed by Alexa Fluor 488-conjugated goat anti-rabbit IgG (1∶1000) and Alexa Fluor 555-conjugated goat anti-mouse IgG (1∶1000) secondary antibodies. Cells were then incubated with Hoechst 33258 (1∶2000), washed thoroughly and mounted on slides using Fluoromount (Sigma, F4680). Confocal cell images were taken using an Olympus IX81 spinning disk confocal microscope.

### Cell fractionation

SCC-13 cells were infected with 10 MOI of tAd5-EV or tAd5-TIG3 and after 24 h the cells were collected and total extract was prepared in cell lysis buffer. A portion of the total extract was set aside for electrophoresis. The remaining extract was centrifuged at 14,000×g and the supernatant and pellet fraction were collected. The pellet fraction was washed once with phosphate-buffered saline and resuspended in gel loading buffer. Total, soluble and pellet fractions were characterized by electrophoresis.

### Antibodies and Reagents

Polyclonal rabbit anti-TIG3 was previously described [23], [55]. BrdU (B2531) and β-actin (A1978) antibodies were purchased from Sigma. Caspase 3 (9665), Caspase 9 (9502), and cleaved PARP (9541) antibodies were from Cell Signaling. Bcl-XL (610211), EEA1 (610456), rab11 (610656), LAMP1 (611042), GM130 (610822), calnexin (610524), and cyclin D1 (554180) antibodies were purchased from BD Transduction Laboratories (San Jose, CA). Bax (sc-493), p21 (sc-6246), cdk4 (sc-601), cdk6 (sc-7181), cyclin E (sc-481), cdk2 (sc-163), cyclin A (sc-239), cyclin B1 (sc-245), and cdk1 (sc-54) antibodies were from Santa Cruz Biotechnology (Santa Cruz, CA). β-tubulin (ab11311), clathrin heavy chain (ab21679), pericentrin (ab28144) and mannose-6-phosphate receptor (ab2733) antibodies were from Abcam (Cambridge, MA). Alexa Fluor 488-conjugated goat anti-rabbit (A-11034), Alexa Fluor 555-conjugated goat anti-mouse (A-21424), and Hoechst 33258 (H3569) were from Invitrogen (Carlsbad, CA). Rabbit HRP-conjugated IgG (NA934) and mouse HRP-conjugated IgG (NXA931) were from GE Healthcare (Piscataway, NJ).

### BrdU Incorporation and flow cytometry

To monitor BrdU incorporation, SCC-13 cells on coverslips were infected with appropriate adenovirus and after 24 h incubated with 10 µM BrdU (BD Pharmingen, 550891) for 2 h at 37 C. Cells were then fixed in 4% paraformaldehyde for 30 min at room temperature and washed with PBS containing 1% Triton X-100. The slides were then incubated in 1 N HCl on ice for 10 min, in 2 N HCl at 25 C for 10 min and 37 C for 20 min. Cells were then incubated with 0.1 M borate buffer for 12 min at 25 C and washed with PBS containing 1% Triton X-100. The cells were blocked in PBS containing 1% Triton X-100, 1 M glycine, and 5% goat serum for 1 h. Sections were incubated with anti-BrdU antibody for 16 h at 4 C. For flow cytometry, SCC-13 cells were infected with tAd5-EV or tAd5-TIG3 and after 24 h floating and attached cells were fixed in methanol, incubated with 20 µg/ml RNase for 30 min at 37 C followed by 50 µg/ml propidium iodide for 1 h prior, and the cells were collected for flow cytometric analysis.

### Real Time-PCR

SCC-13 cells were infected with tAd5-EV or tAd5-TIG3 and at 24–48 h harvested for isolation of mRNA using the illustra RNAspin Mini Isolation Kit (GE Healthcare, 25-0500-71). cDNA was synthesized from isolated RNA using SuperScript III Reverse Transcriptase (Invitrogen, 18080-044) following the manufacturer's protocol. RT-PCR was performed using LightCycler 480 SYBR Green I Master (Roche, 04707516001). RT–PCR primer sequences include: TIG3 (5′-CAGTATTGTGAGCAGGAACTGTGA, 5′-TTGGCCTTTTCCACCTGTTTAC), human cyclophilin (5′-CATCTGCACTGCCAAGACTGA, 5′-TTCATGCCTTCTTTCACTTTGC), and human p21 (5′-AAGACCATGTGGACCTGTCACTGT, 5′-AGGGCTTCCTCTTGGAGAAGATCA).
